# Successful rescue combination of extracorporeal membrane oxygenation, high-frequency oscillatory ventilation and prone positioning for the management of severe methicillin-resistant *Staphylococcus aureus* pneumonia complicated by pneumothorax: a case report and literature review

**DOI:** 10.1186/s12890-017-0445-z

**Published:** 2017-07-20

**Authors:** Hangyong He, Hao Wang, Xuyan Li, Xiao Tang, Rui Wang, Bing Sun, Zhaohui Tong

**Affiliations:** Department of Respiratory and Critical Care Medicine, Beijing Institute of Respiratory Medicine, Beijing Key Laboratory of Respiratory and Pulmonary Circulation, Beijing Chao-Yang Hospital, Capital Medical University, No. 8 Gongren Tiyuchang Nanlu, Chaoyang District, Beijing, 100020 China

**Keywords:** Necrotizing pneumonia, *Staphylococcus aureus*, Panton-valentine leukocidin, Extracorporeal membrane oxygenation, High frequency oscillatory ventilation, Prone position

## Abstract

**Background:**

To describe the experience of combination therapy with extracorporeal membrane oxygenation(ECMO), high-frequency oscillatory ventilation(HFOV) and prone positioning in treating severe respiratory failure caused by community acquired methicillin resistant *Staphylococcus aureus*(CA-MRSA).

**Case presentation:**

A 30-year-old female presented with fever and dyspnea for 3 days. She was diagnosed CA-MRSA pneumonia complicated by severe respiratory failure, pneumothorax and neutropenia. Venovenous ECMO was applied within 8 h of the pneumothorax diagnosis. For amelioration of ventilator-induced lung injury, HFOV and prone positioning were combined with ECMO. The patient’s condition improved considerably. ECMO was weaned on day 19, and she was discharged on day 48 with good lung recovery.

**Conclusions:**

To the best of our knowledge, this was the first case in which ECMO was combined with HFOV and prone positioning to treat severe necrotic CA-MRSA pneumonia complicated with pneumothorax. This combination therapy may provide safe respiratory support, may minimize the risk of barotrauma, and provide better drainage of secretions in patients with necrotizing pneumonia.

## Background

Pneumonia caused by *Staphylococcus aureus* (SA) is usually characterized by lobar consolidation and pneumatoceles, with rapid progression to severe respiratory failure. Barotrauma may lead to pneumothoraces despite a lung-protective ventilation strategy, thus causing more severe hypoxemic respiratory failure and a greater challenge for conventional mechanical ventilation [1]. Community-acquired methicillin-resistant *S. aureus* (CA-MRSA) pneumonia, a community-acquired infection that predominantly affects young people, has a mortality rate > 70% despite aggressive conventional management [2]. Panton-Valentine leukocidin (PVL) is a pore-forming toxin secreted by strains epidemiologically-associated with CA-MRSA and lethal necrotizing pneumonia [3].

Due to the high mortality and frequency of pneumothoraces, in the necrotic lungs of patients with CA-MRSA pneumonia, non-conventional respiratory support strategies, such as extracorporeal membrane oxygenation (ECMO), high-frequency oscillatory ventilation (HFOV), prone positioning, and extracorporeal carbon dioxide removal (ECCO_2_R), have been used [1, 2]. When patients are supported with ECMO and safe oxygenation is reached, HFOV may help re-open the collapsed lung and prone positioning may provide good drainage of necrotic secretions in the lung, which together may lead to rapid recovery and prevent or help heal the pneumothoraces. Although each strategy has been reported independently in previous studies [1, 2], there is very little information in the literature regarding the management of patients with PVL-producing CA-MRSA pneumonia and combination support with ECMO,HFOV, and the prone positioning.

## Case presentation

**Fig. 1:**
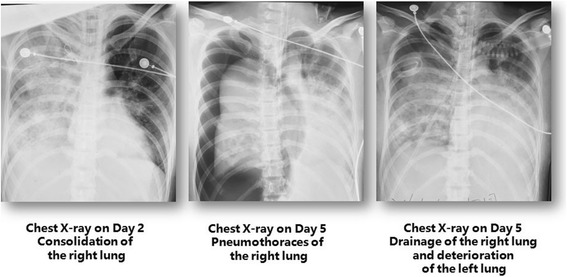
Chest X-rays on day 2 in the outside hospital, on day 5 when right pneumothorax occurred, and on day 5 after a tube was inserted

A 30-year-old female with no medical history was admitted to a local hospital with fever, cough, and dyspnea for 3 days. She was neutropenic with a white cell count of 1.2 × 10^9^/L and the C-reactive protein was 124 mg/L on admission to the outside hospital (from now on considered as day 1 for the case timeline). A Gram stain of sputum revealed MRSA on day 4. Antibiotic therapy included meropenem, moxifloxacin, and teicoplanin.Rapid clinical deterioration prompted intubation and mechanical ventilation (day 2). After 3 days of conventional management with ventilation at a peak inspiratory pressure (PIP) of 30 cm H_2_O, positive end expiratory pressure (PEEP) of 18 cm H_2_O, and a FiO_2_ of 1.0, she developed a right lung pneumothorax and the oxygenation could not be maintained despite a right-sided intercostal chest drain (day 5; Fig. 1). The arterial blood gas was as follows: pH 7.36, PaO_2_ 57 mmHg, PaCO_2_ 39 mmHg. She was referred to our hospital, the Beijing Chao-Yang Hospital Respiratory Intensive Care Unit (RICU), for ECMO support on day 5. Venovenous ECMO (VV-ECMO) was established through the right internal jugular and right femoral vein at the bedside of the local hospital, and she was transferred to the RICU with an ambulance across a distance of 290 km.

Upon arrival at our RICU (day 5), ventilation was at a very low pressure and low FiO_2_ lung protective settings (PIP 10 cmH_2_O, PEEP 0 cmH_2_O, and FiO_2_ 0.4). The diagnosis of PVL-expressing CA-MRSA pneumonia after influenza was confirmed by culture and influenza A nucleic acid PCR during support with ECMO. PVL genes was detected with polymerase chain reaction method, as described in previous reports by Lina et al. [4]. The procalcitonin level was 44 ng/mL; MRSA was also cultured from the right thorax drainage fluid, and empyema was diagnosed. The antibiotic regimen was changed to imipenem/cilastatin, vancomycin (1.0 g every 8 h). Because aspergillosis was reported in influenza patients in recent study [5], and positive galocomannan was detected in her serum and bronchoalveolar fluid samples, voriconazole was used at her admission to our RICU. The serum trough vancomycin levels were monitored two days after its initial dose was given. The trough vancomycin levels range from 4.8 to 8.9 mg/L for 5 days (from day 7 to day 12); vancomycin was thus changed to linezolid on day 13. As *Acinetobacter baumannii* was cultured from her sputum on day 16, tigecycline and cefoperazone/sulbactam was started to used on days 17 and 30, respectively.

**Fig. 2:**
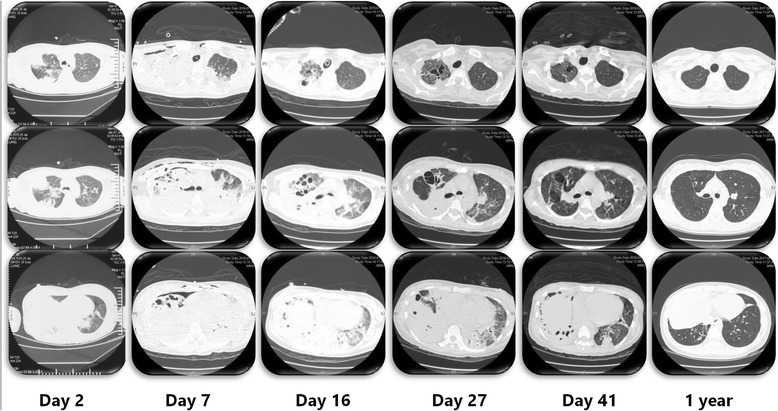
Chest computed tomography (CT) on day 2 before RICU admission and on day 7, day 16, day 27 and day 41 and followed up after 1 year. Three representative slices of the upper, middle and lower lobe were chosen

**Fig. 3:**
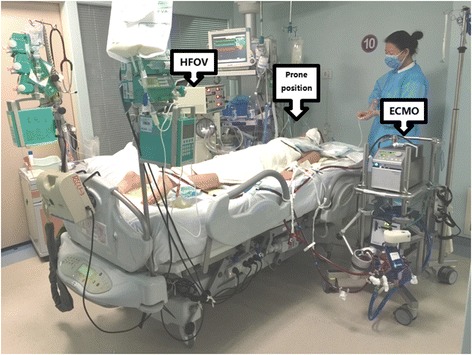
The patients under the combination therapy of ECMO, HFOV and prone position on day 7

**Fig. 4:**
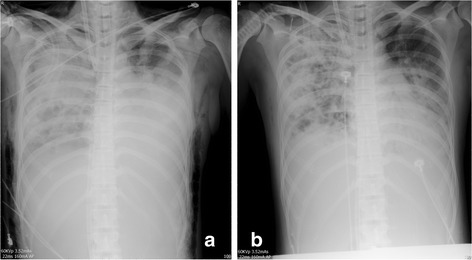
Chest X-ray on day 7 (**a**) before ECMO, HFOV and prone position combination therapy and on day 9 (**b**) after the combination therapy

Chest computed tomography showed bilateral necrotic pneumonia with massive consolidation, especially in the right lung (day 7; Fig. 2). Because of a persistent air leak from the right lung, ventilation was converted to HFOV at a mean airway pressure of 15 cmH_2_O and amplitude of 50 cmH_2_O (day 7). HFOV was used for 23 h per day, and the patient was changed to PCV (PIP of 10 cmH_2_O, PEEP of 0 cmH_2_O) for less than an hour to evaluate her respiratory mechanics, such as tidal volume. Bedside bronchoscopy was performed daily and demonstrated a large amount of necrotic secretions from the left and right main bronchus. The tidal volume was evaluated daily on conventional mechanical ventilation, and showed <100 ml. The patient was deeply sedated, thus a spontaneous cough for sputum was unavailable. Bronchoscopic suction did not fully clear the accumulated necrotic secretion in the bilateral lower lobes. For better drainage of the secretions from the lower lobe of the consolidated lung, the patient was placed in the prone position with ECMO and HFOV(day 6); 8 h daily for 12 days. The prone positioning was arranged by a trained respiratory therapist team with nurses (Fig. 3). The air leak was closed and the tidal volume subsequently improved to 200 ml. The consolidated lung was re-opened by HFOV and the prone position, as evidenced by a chest X-ray 2 days after combination therapy was initiated (day 9; Fig. 4). No adverse and unanticipated events was observed.

**Fig. 5:**
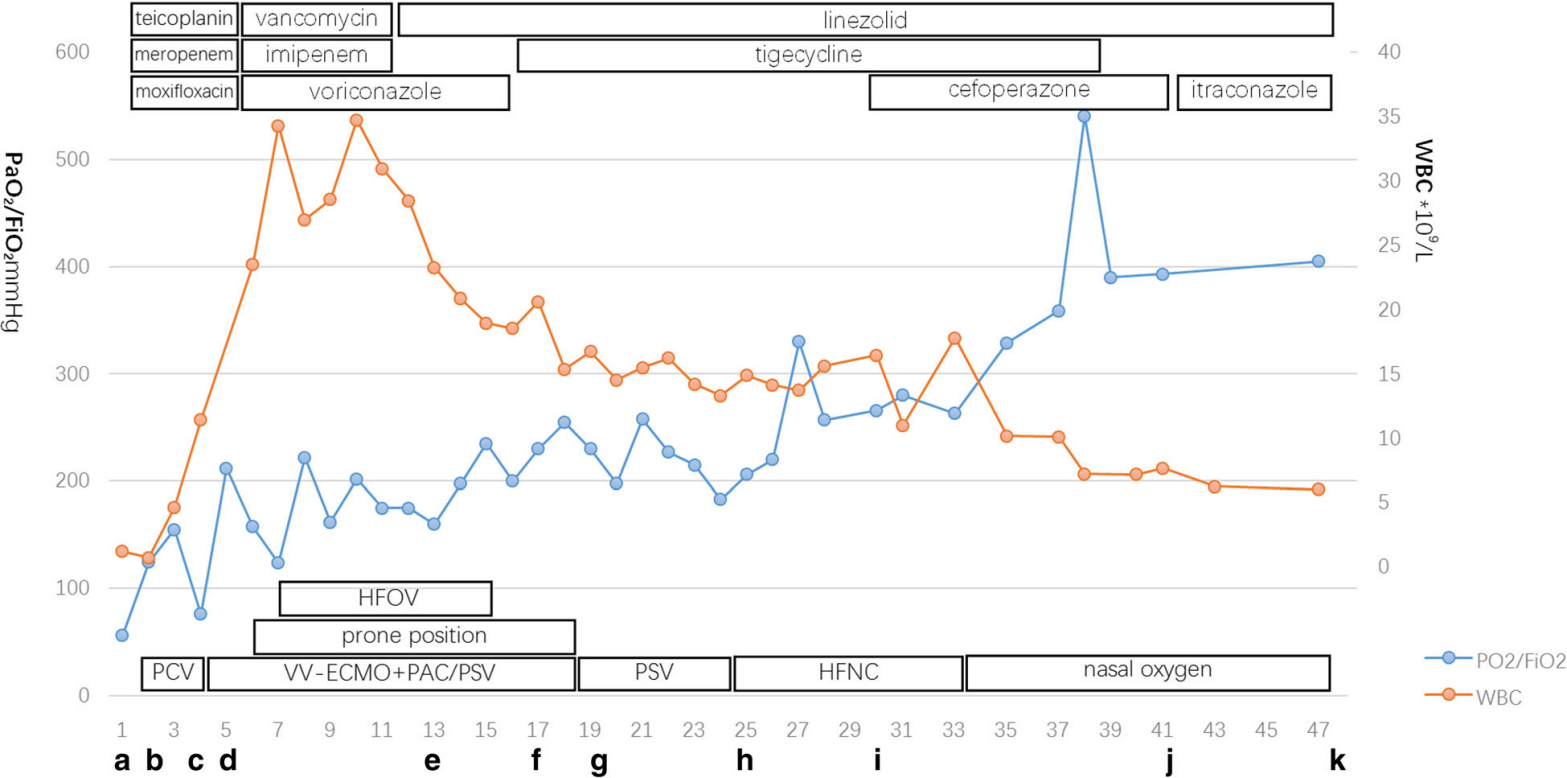
**a**. Day 1, admission in the local hospital. **b**. The patient was intubated and ventilated with PCV on day 2. **c**. The sputum culture yielded MRSA on day 4. **d**. Complicating with right pneumothorax, the patient accepted VV-ECMO to maintain oxygenation and transferred to our RICU for further support. HFOV, prone position and PSV with protective strategy were used for promoting lung recovery and removal of secretion. **e**. On day 13, insufficient vancomycin drug concentration urge the change to linezolid. The chest tube was also pulled out on this day. **f**. Nosocomial *Acinetobacter baumannii* infection was detected, tigecycline was used for strengthening the antibiotic therapy on day 17. **g**. VV-ECMO was weaned on day 19. **h**. The patient was extubated on day 25. **i**. Cefoperazone/sulbactam was used for extensive drug resistant *Acinetobacter baumannii* infection on day 30. **j**. *Candida albicans* infection was detected, itraconazole was added. **k**. The patient was discharged home on day 48. Vancomycin and imipenem: Day 6–12; voriconazole: Day 6–20; linezolid: day 13–48; tigecycline: day 17–39; cefoperazone:day 30–42; itraconazole: day 43–48. Abbreviations: MRSA, methicillin resistant *Staphylococcus aureus*; ECMO, extracorporeal membrane oxygenation; HFNC, high flow nasal catheter; HFOV, high frequency oscillatory ventilation; PSV, pressure support ventilation; PCV, pressure control ventilation

ECMO was weaned on day 19 and mechanical ventilation was weaned on day 25. She was discharged home on day 48. CT scans on days 16, 27, 41, and she was followed up after 1 year of discharge showed a rapid repair of the necrotic lung (Fig. 2). A time line of the events, antibiotics, and ventilation modes is illustrated in Fig. 5.

## Review of cases from the literature

As shown in Table 1, five previously published case series and the current case with PVL-producing CA-MRSA pneumonia supported with ECMO were reviewed. A total of 20 cases were reported, including 4 adults and 16 children or adolescent patients. Pneumothoraces occured in 60% of the patients with severe CA-MRSA pneumonia. ECMO was established in 70% of patients with most severe ARDS. HFOV was combined with ECMO in 3 cases. A lateral position was reported in one case for better drainage, but prone positioning was not reported in combination with ECMO before the current case. The survival rate of the whole case series was 70%, and 64% in patients supported with ECMO.

**Table 1:** Case series reported PVL-MRSA caused ARDS complicated with pneumothorax and supported with ECMO

Citation	Age group	PVL positive (No. of cases)	Intubation (No. of cases)	Pneumothorax (No. of cases)	ARDS	ECMO	HFOV	Prone position	Surgery	Survival	Survival with ECMO
Panchabhai, 2015 [18]	1 adult	1	1	0	0	1	0	Right lung up	1 left pneumonectomy	1/1	1/1
Schwartz, 2012 [19]	4 infants	4	4	3	4	2	NR	NR	0	2/4	0/2
Noah, 2010 [2]	2 adults 2 adolescents	4	4	3	4	4	1	NR	2 thoracotomy	4/4	4/4
Castaldo, 2007 [20]	8 children	NR	7	4	7	4	NR	NR	NR	4/8	1/4
Stroud, 2007 [1]	2 children	NR	2	1	2	2	1	NR	0	2/2	2/2
This case	1 adult	1	1	1	1	1	1	1	0	1/1	1/1
Total	20 patiens 4 adults 16 children/adolescents	100% (10/10)	95% (19/20)	60% (12/20)	90% (18/20)	70% (14/20)	37.5% (3/8)	50% (1/2)	25% (3/12)	70% (14/20)	64% (9/14)

*PVL* Panton-Valentine leukocidin, *MRSA* methicillin resistant *Staphylococcus aureus*, *ARDS* acute respiratory distress syndrome, *ECMO* extracorporeal membrane oxygenation, *HFOV* high frequency oscillatory ventilation, *NR* not reported

## Discussion

PVL-producing CA-MRSA has now been established as a pathogen responsible for a rapidly progressive, frequently fatal disease manifested as necrotizing pneumonia. CA-MRSA pneumonia requires early suspicion, especially in young otherwise healthy individuals with rapidly evolving clinical features, including cavitary consolidation, bilateral infiltrates, pleural effusion, and hemoptysis [6]. Although primary infection can occur in young, healthy, immunocompetent patients, there is increasing evidence to suggest that PVL-MRSA pneumonia may occur secondarily after influenza infection, thus raising concern about possible outbreaks of PVL-MRSA pneumonia during influenza epidemics [7]. Additionally, a number of patients with severe CA-MRSA pneumonia complicated pneumothoraces, can be attributed to barotrauma with a high level of ventilator pressure settings and the underlying pathology of lung necrosis. When single or bilateral pneumothoraces occur, lung function dramatically deteriorates, thus causing refractory hypoxemia and hypercapnia [1]. Indeed, a mortality rate > 70% has been reported after conventional intensive care even though most of the patients were previously healthy, young individuals [6]. As the cases we reviewed, pneumothoraces occurred in 60% of the patients with severe CA-MRSA pneumonia.

VV-ECMO is increasingly recognized as a valuable respiratory support modality in patients with severe ARDS caused by MRSA [8], especially those patients complicated by barotrauma and in need of ultra-protective ventilation to treat the air leak. VV-ECMO support with conventional ventilation, however, has some limitations in the treatment of necrotic pneumonia. First, low pressure, low tidal volume ventilation cannot help the relatively normal lung region re-open. Second, a persistent supine position hinders the drainage of necrotic secretions which have accumulated in the lower lobe. These two factors may lead to prolonged recovery or even a negative outcome. In some centers where ECMO is unavailable, HFOV and prone positioning have been combined for patients with refractory hypoxemia ARDS [9].

In recent years, based on the sufficient oxygenation provided by ECMO, the combination of prone positioning has become feasible in patients with severe ARDS. Guervilly reported that, patients with severe hypoxemia (PaO_2_ to FiO_2_ ratio < 70) despite maximal oxygenation, injurious ventilation parameters with a plateau pressure > 32 cmH_2_O or a failed attempt to wean ECMO after at least 10 days on ECMO support were considered for prone positioning combined with ECMO [10]. Despite repeated bronchoscopic suctioning, effective drainage of necrotic secretions was not possible in our deeply sedated patient on ECMO. Therefore, we propose the prone position by recruiting the dorsal regions of the consolidated lungs and draining the dorsal regions with necrotic secretion accumulation, which could thus exert beneficial effects during ECMO therapy [11]. In our review of literature, 100% of adult patients were treated with ECMO, and only 62.5% of pediatric patients were on ECMO.

Currently, HFOV may still be considered as a rescue therapy option in patients with refractory hypoxemia after the use of ventilation in the prone position, recognizing that prone patients may be oscillated [12]. The combination of HFOV may be useful in patients with persistent severe hypoxemia despite VV-ECMO full support, especially in the 2.4% - 21% of patients with pneumothoraces [1, 2, 13]. Four of 5 reported patients with CA-MRSA pneumonia were supported by ECMO complicated with pneumothoraces, 3 patients had an operative intervention, and 2 patients were changed to HFOV from conventional ventilation [1, 2]. Successful use of HFOV to overcome a persistent air leak with underlying lung collapse and associated refractory hypoxemia eliminating the need for operative intervention has been reported [14–16]. The major problems associated with a persistent air leak in a ventilated patient are persistent lung collapse, ineffective delivery of tidal volume and inability to apply PEEP. The goal of controlling the air leak, reducing the flow from the fistula, and permitting the air leak to heal is not achieved despite the best possible conventional ventilator strategy, thus leading to a refractory air leak and an operative intervention. HFOV generates a high continuous distending pressure, which is reduced from the ventilator to the distal trachea. HFOV prevents the repeated opening–closing phenomenon of alveoli and may recruit collapse of unstable alveolar units more efficiently than conventional ventilation. This mechanism might have played a role, at least in part, in the successful management of our patient [14]. Moreover, HFOV should be viewed only as a rescue maneuver and not as a mainstay in ARDS management. Two recent RCTs on HFOV have shown either neutral or negative results.

Our case has limitations. First, from the first 24–48 h of presentation to the outside hospital, the patient should have been prone and on neuromuscular blocking agents (2 established interventions for severe ARDS). Because the patient was rescued in the local hospital, she was probably not managed ideally from the beginning. Second, the patient did not receive toxin-inhibiting drugs such as clindamycin at the early stage in the local hospital. This two points should be noted, which may lead to her deterioration and pneumothorax. Moreover, in young patients even severe MRSA pneumonias with associated pneumothoraces can recover without ECMO or HFOV was reported [17].

## Conclusions

To the best of our knowledge, this was the first case in which ECMO was combined with HFOV and prone positioning to treat severe necrotic CA-MRSA pneumonia complicated with pneumothorax. This combination therapy may provide safe respiratory support, may minimize the risk of barotrauma, and provide better drainage of secretions in patients with necrotizing pneumonia.

## Abbreviations

ARDS: Acute respiratory distress syndrome
CA-MRSA: Community acquired methicillin resistant *Staphylococcus aureus*
ECMO: Extracorporeal membrane oxygenation
HFOV: High frequency oscillatory ventilation
PEEP: Positive end expiratory pressure
PIP: Peak inspiratory pressure
PVL: Panton-Valentine leukocidin
RICU: Respiratory intensive care unit
